# Heparan Sulfate Proteoglycans as Emerging Players in Synaptic Specificity

**DOI:** 10.3389/fnmol.2018.00014

**Published:** 2018-01-26

**Authors:** Giuseppe Condomitti, Joris de Wit

**Affiliations:** ^1^VIB Center for Brain & Disease Research, Leuven, Belgium; ^2^Department of Neurosciences, KU Leuven, Leuven, Belgium

**Keywords:** heparan sulfate proteoglycans, synapse, circuit assembly, cell surface receptor, connectivity, synapse development, wiring logic, receptor ligand interaction

## Abstract

Neural circuits consist of distinct neuronal cell types connected in specific patterns. The specificity of these connections is achieved in a series of sequential developmental steps that involve the targeting of neurites, the identification of synaptic partners, and the formation of specific types of synapses. Cell-surface proteins play a critical role in each of these steps. The heparan sulfate proteoglycan (HSPG) family of cell-surface proteins is emerging as a key regulator of connectivity. HSPGs are expressed throughout brain development and play important roles in axon guidance, synapse development and synapse function. New insights indicate that neuronal cell types express unique combinations of HSPGs and HS-modifying enzymes. Furthermore, HSPGs interact with cell type-specific binding partners to mediate synapse development. This suggests that cell type-specific repertoires of HSPGs and specific patterns of HS modifications on the cell surface are required for the development of specific synaptic connections. Genome-wide association studies have linked these proteins to neurodevelopmental and neuropsychiatric diseases. Thus, HSPGs play an important role in the development of specific synaptic connectivity patterns important for neural circuit function, and their dysfunction may be involved in the development of brain disorders.

## Introduction

The brain harbors a large variety of neuronal cell types connected by specific patterns of synaptic connectivity. Establishing precisely connected, functional neural circuits requires the guidance of neuronal processes to target areas, the identification of postsynaptic target cells, and the formation of specific types of synapses on defined subcellular compartments of those cells (Sanes and Yamagata, 2009; Shen and Scheiffele, 2010; Williams et al., 2010; Yogev and Shen, 2014). The molecular mechanisms orchestrating this extraordinary synaptic specificity are now starting to be unraveled. Rapid advances in experimental methodologies, such as cell-type specific transcriptome analysis, proteomics, interactome studies and genetics have identified a key role for cell-surface proteins in synaptic specificity (Kolodkin and Tessier-Lavigne, 2011; de Wit and Ghosh, 2016). In this review article, we focus on an ancient class of cell-surface molecules that is emerging as a novel regulator of synaptic specificity: the heparan sulfate proteoglycans (HSPGs). We will first discuss the role of these molecules in different aspects of synapse formation and function, in invertebrate and vertebrate species. We will then consider emerging evidence that supports a role for HSPGs as novel regulators of synaptic specificity in developing neural circuits. Finally, we discuss the implications of perturbations in HSPG expression and biology in neurodevelopmental disorders for the function of neural circuits.

## HSPG Biology

**Figure 1:**
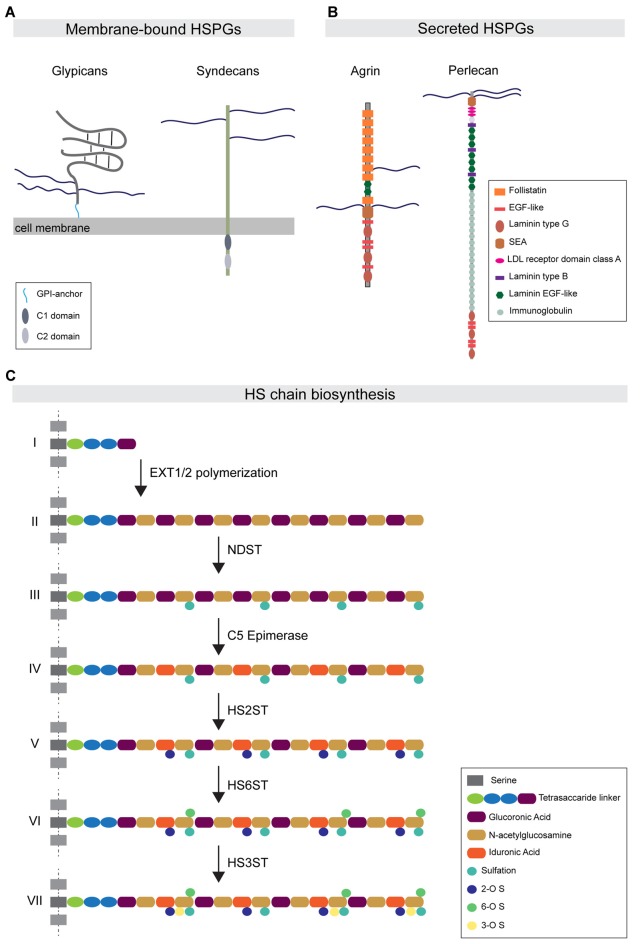
Heparan sulfate proteoglycan (HSPG) protein family organization and HS biosynthesis. **(A)** Major HSPG protein families. Membrane-bound HSPGs can be distinguished in glypicans and syndecans. Glypicans are characterized by a globular protein domain (gray) and a stalk-like domain that contains three attachment sites for HS chains (blue). These molecules are attached to the external leaflet of the cell membrane through a glycosylphosphatidylinositol (GPI)-anchor (light blue). The transmembrane syndecans are characterized by the presence of three HS chains at the N-terminal portion of the protein. The syndecan intracellular tail contains two conserved regions: the C1 domain (dark gray) and C2 (light gray). **(B)** The major secreted HSPGs are Agrin and Perlecan. Agrin carries its HS chains in a central domain and is characterized by the presence of several follistatin domains. Perlecan presents HS chains only at the N-terminal portion of the protein. Its core protein organization is characterized by the presence of multiple conserved domains, such as the immunoglobulin domain. **(C)** The HS chain biosynthetic pathway: (I) HS biosynthesis starts with the attachment of a tetrasaccharide linker to specific serine residues. (II) The EXT family of co-polymerases mediates the elongation of the HS chain by adding disaccharide units composed of glucuronic acid (purple) and N-acetylglucosamine (ocher). (III) Subsequently, the enzyme NDST mediates the simultaneous sulfation and de-acetylation of specific N-acetylglucosamine residues. (IV) The C5-epimerase induces the epimerization of glucuronic acid to iduronic acid (IdoA). (V–VII) The subsequent action of the enzymes HS2ST, HS6ST and HS3ST mediates the attachment of sulfate groups to specific saccharide residues.

HSPGs are cell-surface and secreted proteins consisting of a core domain to which long linear HS glycosaminoglycan chains are covalently attached (Sarrazin et al., 2011). HSPGs function in a wide range of cellular processes by direct interactions with different binding partners. Most of these interactions occur in an HS-dependent and specific manner, with interacting proteins binding to defined structural motifs in the HS chains (Xu and Esko, 2014). Based on their subcellular localization, HSPGs can be grouped into three main subfamilies. The first subfamily consists of the four syndecans (SDC1-4 in vertebrates), which are localized at the cell surface via their transmembrane domain. The second subfamily is represented by the glypicans (GPC1-6 in vertebrates), which are localized at the cell membrane via a glycosylphosphatidylinositol (GPI) anchor (Figure 1A). In addition to syndecans and glypicans, other membrane-associated HSPGs have been identified, such as epican and betaglycan, which also localize to the cell membrane through a transmembrane domain. The third main HSPG subfamily is comprised of the secreted HSPGs agrin, perlecan and collagen type XVIII (Figure 1B). Lastly, a fourth subtype of HSPG has been described: serglycin, which specifically localizes to the luminal side of intracellular vesicles of mast cells and hematopoietic cells (Sarrazin et al., 2011).

These HSPG subfamilies are conserved throughout evolution, from the nematode *Caenorhabditis elegans* and the arthropod *Drosophila melanogaster* to *Homo sapiens*. Vertebrates express multiple members of each HSPG subfamily, whereas invertebrate organisms express fewer members. For instance, the *Drosophila* genome encodes only one copy of syndecan and two copies of glypicans, Dally and Dally-like protein (Dlp; Selleck, 2001). Phylogenetic analysis of HSPG sequences has shown that HSPGs were already present five hundred million years ago in metazoan organisms such as Cnidaria, indicating that HSPGs are ancient molecules (Medeiros et al., 2000; Van Vactor et al., 2006).

Each type of HSPG contains a defined number of HS chains linked to the core protein domain, with syndecans carrying up to five HS chains, while glypicans and secreted HSPGs comprise up to three HS chains. These polysaccharide chains consist of repeated disaccharide units, glucuronic acid and N-acetylglucosamine, which are synthesized in the Golgi apparatus and polymerized onto the core protein through a multistep process that requires the coordinate action of different enzymes (Esko and Selleck, 2002). Following the polymerization steps, the newly synthesized chains undergo a sequential modification process catalyzed by various enzymes. N-deacetylase/N-sulfotransferase (NDST); 2-O-, 3-O- and 6-O-Sulfotransferases (HS2ST1, HS3ST1 and HS6ST1); and C5-Epimerase (GLCE) catalyze deacetylation, sulfation and epimerization reactions, respectively, at the level of specific disaccharide residues (Figure 1C). The combined action of these enzymes differentially affects the composition and properties of the HS chain. NDST activity causes the simultaneous formation of highly sulfated and acetylated subdomains, which form important components of ligand-binding motifs. In addition, the 2-O-, 3-O- and 6-O-sulfotransferase enzymes mediate the addition of sulfate groups only to specific glucosamine residues. Furthermore, two endosulfatases, SULF1 and 2, localize to the plasma membrane and selectively catalyze the removal of 6-O sulfate groups from a subset of trisulfate disaccharide residues in the HS chain (Ai et al., 2006). GLCE-mediated epimerization regulates the conversion of glucuronic acid to iduronic acid (IdoA). As IdoA residues are subsequently sulfated by HS2ST, epimerization is necessary to instruct the positioning of sulfation on the HS chains (Kreuger and Kjellén, 2012).

Importantly, these enzymatic modifications occur in clusters along the HS chain, with short stretches of modified subregions interspersed with long unmodified regions. This heterogeneity of the HS chain is thought to provide specific binding regions that allow the interaction of HS with different protein ligands (Xu and Esko, 2014). HS chain composition is highly regulated, as HS-modifying enzymes show tissue-specific, as well as cell type-specific expression patterns. Furthermore, HS-modifying enzyme expression patterns vary during development (Allen and Rapraeger, 2003; Paul et al., 2017). Within a given cell type, different types of HSPGs contain HS chains with indistinguishable modification patterns and highly similar structural properties (Kato et al., 1994; Tumova et al., 2000; Zako et al., 2003). As the brain is a highly heterogeneous tissue harboring many different cell types, there is an enormous potential for HS diversity. This has led to the hypothesis of a “HS code”, which poses that tissue- and cell type-specific HS modifications control the interaction with particular binding partners in a localized fashion to regulate wiring specificity (Bülow and Hobert, 2004; Holt and Dickson, 2005; Bülow et al., 2008).

## Regulation of Cellular Function by HSPGs

HSPGs were initially described as a component of the extracellular matrix (ECM). Perlecan, agrin and collagen XVIII are indeed found in the extracellular environment of various tissues, where they are important for providing mechanical resistance and for allowing diffusion of molecules throughout the ECM (Bishop et al., 2007). In addition to this structural role, it has become clear that HSPGs are major, and multi-faceted, regulators of developmental signaling, by binding to and modulating the activity of key molecules, such as fibroblast growth factor (FGF), WNT, transforming growth factor (TGFβ) and hedgehog (Hh). One way by which HSPGs regulate these signaling molecules, is by promoting the formation and the maintenance of morphogen gradients. Ablation of HSPGs or of HSPG-biosynthetic enzymes alters the development of these morphogen patterns (Häcker et al., 2005). In addition to this cellular function, HSPGs can also directly act as signaling molecules. Some HSPGs can be enzymatically cleaved and secreted in the extracellular space, where they act as biological effectors. For example, the transmembrane HSPGs SDC1 and SDC4 are cleaved by different types of matrix metalloproteinases in response to several stimuli (Fitzgerald et al., 2000; Park et al., 2000). The released SDC1 ectodomain plays an important role during inflammatory processes (Kainulainen et al., 1998; Li Q. et al., 2002). During acute lung injury, released SDC1 interacts in an HS-dependent manner with the chemokine Cxcl1. The shed SDC1/Cxcl1 complex establishes a chemotactic gradient that guides invading neutrophils to the inflammation site (Li Q. et al., 2002). In addition to a role as signaling molecules, HSPGs can also act in a cell-autonomous way, by functioning on the cell surface as co-receptors for growth factors and their receptors. This is exemplified by the role of HSPGs in the interaction of the growth factor FGF with its receptor (FGFR). HS chains mediate the high affinity binding between FGF and FGFR, and control FGF-mediated signaling during *Drosophila* development (Ornitz, 2000; Schlessinger et al., 2000; Yan and Lin, 2007). In *Drosophila* tracheal morphogenesis, the HSPG Dally-like specifically mediates the interaction between FGF and its receptor Breathless (BTL), and is required to induce a FGF-BTL-mediated signaling cascade (Yan and Lin, 2007). Finally, HSPGs can recruit and cluster cell-surface molecules in membrane domains and regulate their function by promoting their secretion or endocytosis. SDC1 has been shown to be internalized from the cell surface membrane through an endocytic process that is clathrin- and caveolin-independent, but requires actin microfilament polymerization and occurs at the level of lipid rafts (Fuki et al., 2000; Zimmermann et al., 2005). Internalization of SDC1 causes the uptake of the SDC1 binding partners FGFR and β1-integrins, leading to an impairment in cell spreading. SDC1 and its binding partners can be recycled to the cell surface membrane through Arf6-positive vesicles, which restores cell motility (Zimmermann et al., 2005). Thus, endocytosis and recycling of SDC1 and its binding partners regulates their cell surface availability important for normal cell function.

The above examples illustrate the various ways by which HSPGs regulate cellular processes, in an HS-dependent manner. Enzymatic modifications of the HS chains generate HS-specific binding motifs that are important for HSPG–protein interactions and consequently for cellular processes. In cerebellar granule cell precursors for example, the morphogen Sonic Hedgehog (Shh) interacts in an HS-dependent manner with GPC5 to promote precursor cell proliferation. This interaction specifically requires 2-O sulfation modifications on IdoA residues of GPC5‘s HS chains, which are then recognized and bound to the Cardin-Weintraub structural motif of Shh. Downregulation of GPC5 expression levels, or enzymatic removal of GPC5’s 2-O sulfation patterns, severely affects Shh-mediated signaling (Witt et al., 2013).

Lastly, in addition to HS-dependent binding to proteins, which form the majority of HSPG interactions, direct binding of signaling molecules to the HSPG protein core has also been described, such as the interaction of the morphogen Hh with the core domain of GPC3 (Capurro et al., 2008). The GPC3-Hh complex is internalized in the cell and directed for degradation in the lysosomal compartment, indicating that GPC3 controls developmental signaling processes by acting as a negative regulator of Hh-mediated signaling (Capurro et al., 2008). Altogether, these examples demonstrate how HSPGs represent a highly diverse and versatile protein family important for regulating a broad range of cellular functions. In the next sections, we will discuss how HSPGs play an important role during brain development as regulators of the various steps leading to synaptic specificity.

## HSPGs as Regulators of Axon Guidance

HSPGs and HS-specific modifications play an important role in the formation of neural connectivity. HSPGs are important regulators of axon guidance, a first key step in the assembly of specific synaptic connections. Here, we provide a brief summary of the roles of HSPGs in this process, as the cellular and molecular mechanisms by which HSPGs regulate axon guidance and targeting have been extensively reviewed elsewhere (Lee and Chien, 2004; de Wit and Verhaagen, 2007). Pioneering studies in cultured cockroach embryos and in *Xenopus laevis* retinal ganglion cells (RGCs) demonstrated that treatment with exogenous HS or enzymatic degradation of HS chains impaired axonal growth and guidance (Wang and Denburg, 1992; Walz et al., 1997). These initial observations were subsequently confirmed in a mouse model in which HS was removed through the conditional deletion of the enzyme Exostosin 1 (EXT1), the key enzyme in HS chain biosynthesis. Loss of EXT1 causes severe axon guidance errors of the major commissural axon tracts (Inatani et al., 2003), indicating that HSPGs are important regulators of axon guidance. Subsequent studies demonstrated that HSPGs control axon guidance through the binding and regulation of different axon guidance cues. For example, HSPGs interact with members of the Slit protein family and promote the binding to their receptor Robo in order to induce Slit-mediated repulsive function (Hu, 2001; Steigemann et al., 2004; Hussain et al., 2006). Furthermore, HSPGs positively regulate the attractive function of the transmembrane guidance molecule Semaphorin 5 (Kantor et al., 2004). Finally, binding of the membrane-anchored guidance molecule Ephrin A3 to HS chains is required for mediating Ephrin A3-induced axon repulsion (Irie et al., 2008). In addition to the general role for HS chains in axon guidance, experimental evidence from different model organisms has also shown the involvement of specific HS enzymatic modifications in this process. Multiple studies in which specific HS-modifying enzymes were deleted in a cell type-specific manner, found different, yet specific axonal targeting defects (Bülow and Hobert, 2004; Bülow et al., 2008; Tillo et al., 2016). In *C. elegans*, ventral D-type motorneuron axons initially grow and fasciculate along the right ventral cord, then cross the midline and on the contralateral side project to the dorsal side of the animal. Bülow and Hobert (2004) demonstrated that each of these steps is differentially affected by the removal of the *C. elegans* homologs of 6-O sulfotransferase (hst-6), 2-O sulfotransferase (hst-2) and the C5-epimerase (hse-5). In particular loss of hse-5 and hst-2 severely impairs axonal fasciculation and dorsal projection (Bülow and Hobert, 2004). However, the loss of hst-6 only affects midline crossing, suggesting that the different steps of D-type motorneuron axon growth require specific HS modification patterns. Strikingly, when the same enzymes were ablated in a distinct type of motorneurons, the DA motorneurons that make similar axon guidance choices, no major defects were observed (Bülow and Hobert, 2004), indicating that cell-type specific HS modification patterns control axon guidance. Altogether, these studies demonstrate that HSPGs and cell type-specific HS chain modification patterns are important regulators of axonal growth and targeting.

## HSPGs as Regulators of Synapse Development

Studies in different model organisms, from *C. elegans* to mouse, have demonstrated that different HSPGs and HS-modifying enzymes regulate multiple aspects of synapse development. In the following section, we highlight the roles of the HSPG protein family in controlling general synapse formation, composition and function, before turning to emerging evidence for a role of HSPGs in synaptic specificity.

### Modulating Localization of Synaptic Signaling Molecules

**Figure 2:**
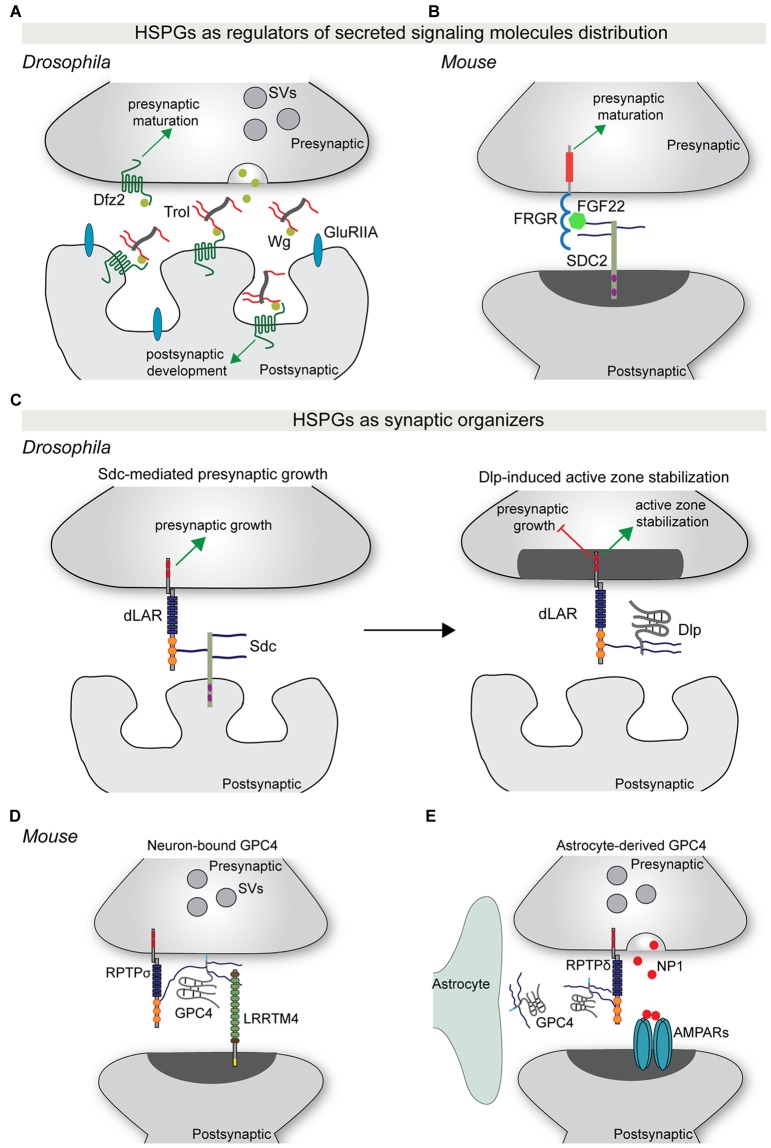
HSPGs as regulators of synapse development.** (A)** The secreted HSPG trol regulates Wg-mediated synaptic differentiation at the *Drosophila* neuromuscular junction (NMJ). Trol is released in the synaptic cleft from the postsynaptic muscle cell. Here, trol binds and sequesters Wg to the surface of the postsynaptic compartment, allowing the interaction with its postsynaptic receptor Dfz2 and the induction of postsynaptic structural and functional maturation. At the same time, Wg also acts presynaptically in a trol-independent manner to instruct presynaptic maturation. **(B)** During vertebrate synapse development, postsynaptic SDC2 binds FGF22 in an HS-dependent manner and facilitates its presentation to the presynaptic receptor FGFR. This interaction promotes presynaptic differentiation. **(C)** HSPGs act as synaptic organizers. During *Drosophila* NMJ development, postsynaptic Sdc promotes presynaptic growth through binding to the presynaptic RPTP dLAR. Subsequently, Dlp, which has a higher affinity for dLAR, competes with Sdc-dLAR binding to inhibit presynaptic growth and promote active zone stabilization. **(D)** GPC4 acts as a presynaptic binding partner for the postsynaptic adhesion protein LRRTM4. GPC4-LRRTM4 interaction occurs in an HS-dependent manner and forms a *trans-*synaptic complex that regulates the development of excitatory synapses. This complex requires RPTPσ, which acts as a presynaptic GPC4 *cis-*receptor to mediate presynaptic development and function. **(E)** During early postnatal mouse visual system development, astrocytes release GPC4. GPC4 binds presynaptic RPTPδ, most probably in an HS-dependent manner, and instructs presynaptic release of NP1, which clusters AMPARs and promotes the formation of active synapses. Abbreviations: SV, synaptic vesicles; SDC2, syndecan 2; Dlp, Dally-like protein; NP1, neuronal pentraxin 1; GPC4, glypican 4.

Secreted synaptogenic molecules, such as WNT and FGFs, are important regulators of synapse formation and maturation (Siddiqui and Craig, 2011). These molecules can be released from the pre-or postsynaptic compartment, or from neighboring astrocytes, and promote synaptic differentiation through binding to their receptors on the neuronal membrane. Recent studies have started to shed light on the role of HSPGs in controlling the synaptic localization of specific secreted synaptogenic molecules. Terribly reduced optic lobe (trol), the *Drosophila* ortholog of perlecan, is secreted by the postsynaptic muscle cells and accumulates in the synaptic cleft (Kamimura et al., 2013). *Trol* mutants show an overproduction of boutons, as well as a reduction in the subsynaptic reticulum area and in glutamate receptor content (Kamimura et al., 2013). Presynaptic organization and composition were unaffected however. The structural defects observed in *trol* mutants are similar to the effects observed in *Wg* mutants, a *Drosophila* homolog of WNT. Indeed, loss of trol causes a reduction in the extracellular levels of Wg, and in particular in its localization in proximity to the postsynaptic compartment. This suggests that the secreted HSPG trol regulates the distribution and localization of Wg at the fly neuromuscular junction (NMJ), thus mediating structural and ultrastructural maturation of the postsynaptic compartment (Kamimura et al., 2013; Figure 2A). In vertebrates, postsynaptic SDC2 interacts with the secreted protein FGF22 in an HS-dependent manner to present FGF22 to the presynaptic FGF receptor, driving bidirectional synaptic maturation (Hu et al., 2016; Figure 2B).

### Organizing the Synaptic ECM

Synapses are enwrapped by a layer of ECM (Frischknecht and Gundelfinger, 2012), which is important for shaping and maintaining synaptic morphology and function. Important components of the ECM are the secreted HSPGs collagen type XVIII, perlecan and agrin (Barros et al., 2011). However, whether and how these secreted molecules contribute to the structural organization of the ECM and synapse development is largely uncharacterized. Evidence for a role of secreted HSPGs in controlling ECM organization and synapse development comes from studies on the *C. elegans* NMJ. Mutant worms for *emb-9* and *cle-1*, the orthologs of collagen type IV and XVIII, have ectopic presynaptic terminals, suggesting that these two molecules are required to restrict the growth of presynaptic boutons (Qin et al., 2014). The growth of ectopic boutons upon loss of Emb-9 and Cle-1 is partially explained by a fragmentation of the basal membrane surrounding the presynaptic bouton, which may favor the formation of ectopic presynaptic terminals (Qin et al., 2014). Interestingly, the ectopic presynaptic terminal growth and ECM defects observed in *emb-9* mutants are reverted by the simultaneous ablation of *unc-52*, the *C*. *elegans* ortholog of Perlecan, indicating that secreted perlecan promotes bouton growth (Qin et al., 2014). These observations suggest that collagens and perlecan differentially regulate synapse development, by acting either as synapse growth-restricting or -promoting factors, respectively. Thus, different secreted HSPGs act simultaneously to control ECM organization and synapse growth. The molecular mechanisms underlying this differential capacity are currently unknown, but it has been previously demonstrated that secreted HSPGs differently control the biomechanical properties of the ECM during *Drosophila* development (Pastor-Pareja and Xu, 2011). The accumulation of collagen type IV causes a more rigid ECM, while perlecan antagonizes collagen IV’s effect, leading to a more elastic extracellular environment (Pastor-Pareja and Xu, 2011). Thus, it seems plausible that at the worm NMJ, the loss of collagen type IV may create a more elastic environment permissive for ectopic bouton growth. In addition to changes in biomechanical properties however, the different ECM composition might also affect the distribution of secreted molecules that regulate synapse development. Whether in addition to secreted HSPGs, other types of HSPGs contribute to organizing the synaptic ECM, and whether HS-specific modification patterns regulate this process is still unknown.

### HSPGs as Synaptic Organizing Molecules

Important regulators of synapse assembly and maturation are the synaptic organizing proteins (Takahashi and Craig, 2013; Ko J. et al., 2015; Jang et al., 2017). These proteins localize to the pre- and postsynaptic membrane, or are secreted in the synaptic cleft, and induce the differentiation of the pre-and postsynaptic element by recruiting components of the synaptic machinery. Synaptic organizing proteins include neurexins (Graf et al., 2004), neuroligins (Scheiffele et al., 2000), leucine-rich repeat transmembrane neuronal proteins (LRRTMs; de Wit et al., 2009; Ko et al., 2009; Linhoff et al., 2009), FGFs (Fox et al., 2007; Terauchi et al., 2010) and thrombospondins (Christopherson et al., 2005; Eroglu et al., 2009). Many additional synaptic organizing proteins have been identified (Siddiqui and Craig, 2011; Um and Ko, 2013; Ko J. et al., 2015; Jang et al., 2017). The first neural synaptic organizer identified was the secreted HSPG agrin (Godfrey et al., 1984; Gautam et al., 1996; Glass et al., 1996). Agrin is secreted by the presynaptic compartment of motorneurons and localizes to the synaptic cleft, where it instructs NMJ postsynaptic differentiation. Loss of agrin causes a reduction in acetylcholine receptor content, a decrease in postsynaptic membrane size and fragmentation of the basal lamina of the synaptic cleft (Gautam et al., 1996). Agrin-mediated postsynaptic organization occurs through the ability of agrin to bind, cluster and activate the postsynaptic tyrosine kinase receptor MuSK (Glass et al., 1996).

More recently, syndecans and glypicans have been identified as synaptic organizers. At the *Drosophila* NMJ, Sdc is expressed by muscle cells and postsynaptically localized, while Dally-like (Dlp) localizes to the perisynaptic space. Both HSPGs regulate different aspects of NMJ synapse formation and function (Johnson et al., 2006; Nguyen et al., 2016). Sdc promotes presynaptic bouton growth, whereas Dlp restricts presynaptic active zone morphogenesis (Johnson et al., 2006). The differential regulation of synaptic architecture by Sdc and Dlp is reflected at the functional level, with loss of *Dlp*, but not of *Sdc*, causing an increase in neurotransmitter release (Johnson et al., 2006). Remarkably, Sdc and Dlp interact with the same presynaptic receptor, the protein tyrosine phosphatase receptor (RPTP) Dlar. Sdc and Dlp bind Dlar at overlapping sites and in an HS-dependent manner. Dlp has a greater affinity for Dlar and effectively competes with Sdc for Dlar binding (Johnson et al., 2006; Figure 2C). Furthermore, Dlp inhibits Dlar signaling, but how Dlar can discriminate between the two HSPGs to instruct differential effects on presynaptic bouton morphology and function remains unclear.

Although these experiments at the fly NMJ indicate that Sdc and Dlp mainly act presynaptically, the cellular source of Dlp is not entirely clear. Experiments in vertebrates have shown that neuron-, as well as glial-derived glypicans play an important role in synapse development. Two independent studies identified HSPGs, and in particular glypican 4 (GPC4), as presynaptic binding partners for the postsynaptic adhesion protein LRRTM4 (de Wit et al., 2013; Siddiqui et al., 2013). GPC4 binds LRRTM4 via its HS sugar chains to form a *trans-*synaptic complex that organizes excitatory synapse development through the clustering of pre- and postsynaptic components (de Wit et al., 2013; Siddiqui et al., 2013). The LRRTM4-GPC4 complex requires presynaptic RPTPσ, which acts as an HS-dependent *cis-receptor* for GPC4 on the presynaptic membrane, to instruct presynaptic development and function (Ko J. S. et al., 2015; Figure 2D).

In addition to a role in the presynaptic neuron, *Gpc4* and *Gpc6* are also expressed and secreted by astrocytes in the early stages of postnatal development. Soluble GPC4 and GPC6 induce excitatory synapse formation in cultured retinal ganglion cells (RGCs) and GluA1-containing glutamate receptor clustering (Allen et al., 2012). Astrocyte-derived GPC4 binds to presynaptic RPTPδ and RPTPσ and induces release of the glycoprotein neuronal pentraxin 1 (NP1) from the presynaptic compartment, which subsequently clusters postsynaptic GluA1-containing glutamate receptors (Farhy-Tselnicker et al., 2017). Interfering with RPTPδ or RPTPσ-mediated signaling blocks GPC4-induced NP1 release and synapse formation. *In vivo*, astrocyte-specific *Gpc4* deletion in the RGC target region, the superior colliculus and *Rptpδ* ablation in both RGCs and the superior colliculus, caused a reduction in synapse formation (Farhy-Tselnicker et al., 2017; Figure 2E).

The studies described above in fly and vertebrate systems demonstrate that presynaptic RPTPs form a central hub to mediate HSPG-induced synaptic development. Whether RPTPs can distinguish GPC4 derived from neurons or glia, and whether GPC4 from different cellular sources would have differential functional effects, is currently unknown. Furthermore, whether glial cells can act as source of GPC4 during later developmental stages and in adulthood is an intriguing possibility that remains to be addressed.

The ability of HSPGs to control synapse development requires an interaction with specific co-receptors in the case of glypicans, which lack a cytoplasmic domain, but can also be accomplished by direct activation of specific intracellular signaling cascades in the case of transmembrane syndecans. In cultured hippocampal neurons, postsynaptic SDC2 clusters in dendritic spines concomitantly with dendritic spine maturation. Overexpression of SDC2 in immature neurons accelerates the development of mature dendritic spines (Ethell and Yamaguchi, 1999). SDC2’s capacity to trigger spine morphogenesis is dependent on its intracellular region. The tyrosine kinase receptor EphB2 phosphorylates SDC2 at tyrosine residues Y281 and Y189 and these two modifications are necessary for SDC2 spine clustering and for triggering spine morphogenesis (Ethell et al., 2001). SDC2 interacts with additional intracellular binding partners, such as syntenin, calcium/CaM-dependent serine protein kinase (CASK), synbindin and synectin (Hsueh et al., 1998; Chen et al., 2011), and the negative regulator of the Ras signaling pathway neurofibromin (Lin et al., 2007). This suggests that SDC2’s interaction with specific scaffolding and signaling proteins regulates dendritic spine maturation.

## HSPGs in Synaptic Specificity

Recent technological advances are accelerating the discovery of the molecular principles underlying synaptic specificity. Genomic, proteomic and interactomic analyses, even at the level of single neurons, are enabling the identification and characterization of classes of cell-surface molecules that might be required for synaptic specificity. To be able to instruct the development of specific synaptic connectivity patterns, these molecules should have several characteristics: they should be expressed in a brain region- and cell type-specific manner; they should be able to interact with distinct and region-specific binding partners; and they should have enough molecular diversity in order to confer cell type- and possibly even synapse type-specific identities (de Wit and Ghosh, 2016). Recent work has started to reveal that, in addition to their role as synaptic organizers, HSPGs show highly specific expression patterns; interact with diverse, region-specific interactors; and also carry synapse-specific modification patterns, suggesting that HSPGs can act as regulators of synapse specificity.

### Cell Type-Specific Expression Patterns of HSPGs and HS-modifying Enzymes

**Figure 3:**
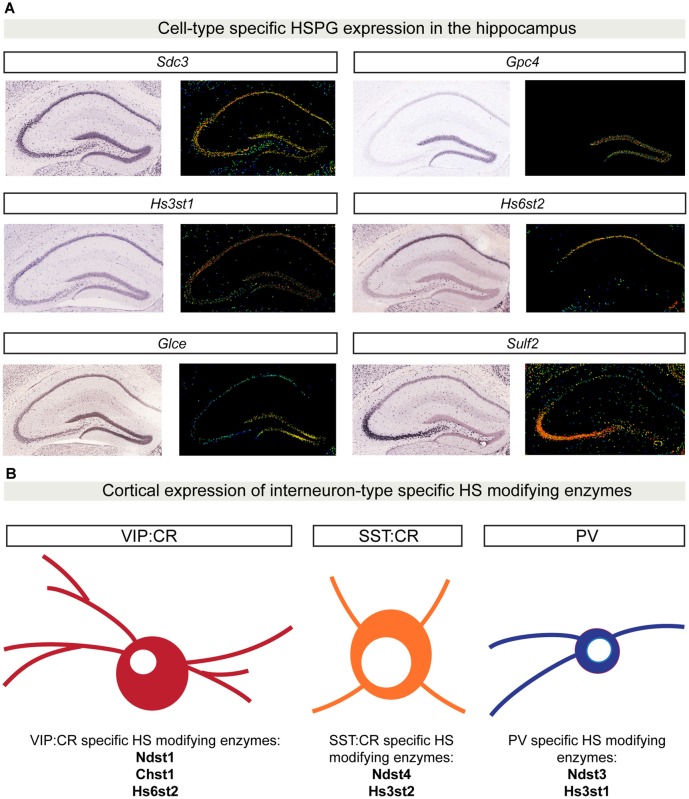
Cell type-specific HSPG and HS-modifying enzyme expression patterns. **(A)** *In situ* hybridization showing gene expression patterns in P56 mouse coronal hippocampal sections for a limited set of HSPGs and HS-modifying enzymes. *Sdc3* (http://mouse.brain-map.org/gene/show/20731) is broadly expressed in the hippocampus, whereas *Gpc4* (http://mouse.brain-map.org/gene/show/14511) is highly enriched in the dentate gyrus (DG) region. The 3-O sulfotransferase *Hs3st1* (http://mouse.brain-map.org/experiment/show/2305) is mainly expressed in CA1 and DG, while *Hs6st2* (http://mouse.brain-map.org/experiment/show/72129255) is specifically expressed only in CA1. *Glce* (http://mouse.brain-map.org/experiment/show/74641306) is specifically expressed in DG, while *Sulf2* (http://mouse.brain-map.org/experiment/show/72007935) expression is highly enriched in CA3. All images in **(A)** are obtained from the Allen Brain Atlas (http://www.brain-map.org/). For each gene, left panel shows the original *in situ* hybridization signal; right panel shows a heat map color scale to indicate intensity of expression. **(B)** Cell type-specific expression patterns for HS-modifying enzymes in distinct cortical interneuron populations. Using single-cell RNA sequencing in six different populations of genetically labeled and phenotypically characterized GABAergic neurons, Paul and colleagues showed a cell type-specific expression pattern of different HS-modifying enzymes. In **(B)**, examples of three different GABAergic isolated populations are shown: vasoactive intestinal peptide (VIP) CR positive GABAergic cells are characterized by the expression of *Ndst1, Chst1* and *Hs6st2*. SST:CR GABAergic cells are characterized by the expression of *Hs3st2* and *Ndst4*. PV positive cells specifically express the enzymes *Hs3st1* and *Ndst3*. Abbreviations: SDC3, syndecan 3; GPC4, glypican 4; Hs3st1, 3-O sulfotransferase type 1; Hs6st2, 6-O sulfotransferase type 2; Glce, C5-epimerase; Sulf2, sulfotransferase 2; Chst1, sulfotransferase 1; Ndst1, N-deacetylase and N-sulfotransferase 1; Hs3st2, 3-O sulfotransferase type 2; Ndst4, N-deacetylase and N-sulfotransferase 4.

An important property for molecules involved in synaptic specificity is region- and cell type-specific expression. Recent advances in single-cell sequencing demonstrate that several synaptic molecules that play an important role in synapse formation are expressed in the brain in a cell type-specific manner (Tan et al., 2015; Földy et al., 2016; Shekhar et al., 2016; Li et al., 2017; Paul et al., 2017). Syndecans and glypicans also show discrete expression patterns in the mouse hippocampus (Figure 3A). Initial *in situ* hybridization studies revealed that syndecans have different, moderately overlapping expression patterns in adult rat brains. *Sdc1* is mainly expressed in the cerebellum, while *Sdc2* and *Sdc4* are enriched in the granule cells of the dentate gyrus (DG) and glial cells, respectively (Hsueh and Sheng, 1999). Glypicans show highly specific expression patterns. *Gpc1*, *2* and *4* are expressed in the hippocampus. *Gpc1* is highly enriched in CA3 pyramidal neurons, while *Gpc2* and *4* are abundant in DG granule cells (with an enrichment of *Gpc4* also in CA1 pyramidal neurons; de Wit et al., 2013; Ko J. S. et al., 2015). Interestingly, the expression pattern of some glypicans changes during development (Ko J. S. et al., 2015). These cell type-specific expression profiles are supported by gene expression profile analysis of the principal hippocampal neuron populations by RNA sequencing (Cembrowski et al., 2016). In addition, recent single-cell RNA sequencing studies have further characterized cell type-specific expression patterns for HSPGs. Li et al. (2017) demonstrated that in the *Drosophila* olfactory bulb, the VM2 projection neurons (VM2-PN) are specifically characterized by the expression of the HSPG *trol*. Furthermore, single-cell transcriptomic analysis of different GABAergic populations in the mouse primary visual cortex has identified a vasoactive intestinal peptide (VIP)-positive interneuron subpopulation that localizes to deep cortical layers and is characterized by the expression of *Gpc3* (Tasic et al., 2016). Altogether, these results indicate that HSPGs have brain region- and cell type-specific expression patterns, supporting a possible involvement in synaptic specificity.

Interestingly, the brain region- and cell type-specificity is not restricted to HSPGs; different HS-modifying enzymes show discrete expression patterns in the brain as well. In the adult mouse brain, the extracellular 6-O-endosulfatases SULF1 and 2 show different expression profiles, with *Sulf2* being broadly expressed, while *Sulf1* is restricted to defined cell layers, such as cortical layer V and the Purkinje cell layer of the cerebellum (Kalus et al., 2009; Figure 3A, hippocampal expression analysis). Single-cell RNA sequencing of six different populations of genetically labeled and phenotypically characterized GABAergic neurons demonstrated cell type-specific expression patterns of sulfotransferases and a layer-specific distribution in adult mouse cortex (Paul et al., 2017; Figure 3B). In addition, by generating a panel of different HS-specific single chain variable fragment antibodies, Attreed et al. (2012, 2016) have shown that in the *C. elegans* central nervous system, distinct cell types present unique HS epitopes on their surface. Furthermore, their results hint at synapse-specific HS modification patterns (Attreed et al., 2016). Together, these findings suggest that different cell types, and possibly different synapse types, display a distinct composition of HSPGs and a specific pattern of HS modifications on their surface, which may be required for the development of precise synaptic connectivity.

### Cell Type-Specific HSPG Binding Partners

In addition to cell type-specific expression patterns, molecules involved in the development of precise synaptic connectivity patterns may interact with region- and cell type-specific binding partners. HSPGs have been shown to interact with synaptic binding partners that are highly restricted to specific brain regions or cell types. As previously described, GPC4 regulates excitatory synapse formation through a *trans-*synaptic interaction with the postsynaptic protein LRRTM4 (de Wit et al., 2013; Siddiqui et al., 2013). In the hippocampus, *Lrrtm4* is only expressed in DG granule cells, while *Gpc4* is broadly expressed in hippocampus and cortex (de Wit et al., 2013; Ko J. S. et al., 2015). Loss of LRRTM4 specifically affects synapse number, function and composition in granule cells, while CA1 pyramidal neurons are unaffected (Siddiqui et al., 2013). As *Gpc4*, and other glypicans, are expressed in different brain regions, these observations suggest that glypicans might interact with different binding partners in other parts of the brain. The extracellular interactomes for glypicans and syndecans have not yet been elucidated.

### HS Modification in Synapse Development

Recent studies have started to explore whether HS chain modifications play a role in synapse development. Using an RNAi-based screen in *Drosophila*, Dani et al. (2012) found that Sulf1 and Hs6st differentially affect synapse composition and function at the NMJ. Loss of *Sulf1* or *Hs6st* causes an increased number of synaptic boutons, but has differential effects on synaptic transmission. *Sulf1* mutants show an increased strength of synaptic transmission, while *Hs6st* mutants have weaker neurotransmission (Dani et al., 2012). Loss of these HS-modifying enzymes affects the synaptic levels of Dlp and Sdc, but do not simply phenocopy the effects of Dlp and Sdc loss (Johnson et al., 2006). *Hs6st* loss reduces Dlp levels, while *Sulf1* removal increases the levels of both Sdc and Dlp. Altered synaptic levels of Dlp and Sdc impair anterograde Wg and retrograde Gbb *trans-*synaptic signaling, important to instruct pre- and postsynaptic maturation (Dani et al., 2012). These findings suggest that a combinatorial function of HSPGs and HS chain modifications mediate synapse formation, function and composition.

Lastly, it is emerging that also in vertebrates, specific HS modification patterns can instruct synapse formation and function. In the CA1 region of the hippocampus, loss of SULF1 specifically causes a reduction in dendritic spine density and impairment in synaptic plasticity, while SULF2 removal does not cause any synaptic structural and functional effects (Kalus et al., 2009). These findings suggest that patterns of HS modification are also required to specify structural and functional synaptic properties in the vertebrate system.

## HSPGs and Disease

The importance of synaptic organizing proteins in normal synaptic function, and therefore proper brain activity, is highlighted by the fact that several recent large-scale genomic analyses have revealed a correlation between genes encoding synaptic proteins and brain disorders (Parikshak et al., 2013; De Rubeis et al., 2014). Recent work has started to shed light on the contribution of HSPGs and of HS-modifying enzymes in neurodevelopmental and neuropsychiatric disorders. A need for HSPGs in proper brain function has initially been demonstrated by Irie et al. (2012). Taking advantage of a conditional knockout mouse for the HS-polymerizing enzyme EXT1, the authors abolished HSPG expression in postnatal excitatory neurons. Postnatal loss of EXT1 did not cause any brain morphological defects, but resulted in autism-like behavioral phenotypes (Irie et al., 2012). The behavioral defects were accompanied by impaired glutamatergic transmission (Irie et al., 2012). Genetic analysis on patients affected by hereditary multiple exostosis and autism-associated mental retardation has identified deletion mutations in the gene encoding EXT1 (Li H. et al., 2002). These observations indicate that neuron-specific HSPG loss recapitulates important aspects of autism pathogenesis, further underscoring the importance of these molecules in normal brain function.

Among all the HSPGs, alterations in glypicans expression have been frequently found in different neuropathological conditions, such as autism spectrum disorders (ASDs), schizophrenia and neuroticism (Potkin et al., 2009; Calboli et al., 2010; Pinto et al., 2010; Doan et al., 2016). For instance, in a genome-wide study in autism patients to identify novel copy number variations (CNVs), four independent CNVs in the *GPC5*/*GPC6* gene cluster were identified (Pinto et al., 2010). Furthermore, Doan et al. (2016) have recently characterized novel human accelerated regions (HARs) in the *GPC4* genomic locus. HARs are human genomic sequences that are conserved in vertebrate evolution, but that are highly divergent in humans. Interestingly, HARs are particularly enriched in genes expressed in the central nervous system (Kamm et al., 2013), and based on their high frequency of mutation, HARs are considered as important genomic elements in the development of human-specific traits (Franchini and Pollard, 2017). Novel HARs have been identified within the *GPC4* genomic locus. In addition, two cases of ASD and intellectual disability (ID) present point mutations in HARs within the *GPC4* locus leading to decreased *GPC4* expression (Doan et al., 2016). These data indicate that regulated *GPC4* expression in the human brain is important for normal central nervous system development. Lastly, in another genome-wide association study to identify common genetic risks that underlie ASDs, a single nucleotide polymorphism that associated with the disease has been identified in the gene encoding the HS 3-O sulfotransferase *HS3ST5* (Wang et al., 2009). Although the molecular mechanisms that link alterations in HSPG and HS-modifying enzyme expression to disease development are still unknown, these findings further strengthen the requirement of HSPGs and HS-modifying enzymes in normal brain development and function.

## Conclusions and Future Directions

The formation of specific synaptic connectivity patterns is a key step in the assembly of functional neural circuits. This process depends on diverse molecules that are expressed in a cell type-specific manner, interact with distinct region- and cell type-specific binding partners, and instruct synapse-specific properties. The HSPG protein family is emerging as an important regulator of synaptic specificity. HSPGs are synaptic organizers and are expressed in a brain region- and cell type-specific manner. HSPGs interact with binding partners expressed in discrete cell types, and through particular HS chain modification patterns exert differential effects on synaptic function. However, many challenges remain in order to elucidate the role of HSPGs in the development of specific synaptic connectivity patterns.

Evidence in support of cell type-specific HSPGs expression patterns comes from recent single-cell transcriptomic analysis of different projection neuron types in the *Drosophila* olfactory bulb and distinct GABAergic populations in the adult mouse cortex that revealed a cell type-specific expression patterns of distinct HSPGs and HS-modifying enzymes (Tasic et al., 2016; Li et al., 2017; Paul et al., 2017). It will be important to determine whether cell type-specific combinations of HSPGs and HS-modifying enzymes broadly exist throughout the brain, and to experimentally address whether these combinations can mediate the formation of specific synaptic contacts and the development of their particular structural and functional properties.

The extent to which different HSPGs interact with region-specific binding partners is also not fully understood. Large-scale interactome screening efforts may accelerate the characterization of the extracellular interactome of different HSPGs and can determine whether these interactions are mediated by the HS chains or the core proteins (Özkan et al., 2013). A greater challenge will be to determine to what extent such interactions are modulated by HS modifications. In combination with expression and protein distribution analysis, region-specific HSPG-ligand interactions can then be tested for a role in instructing the assembly of specific synaptic connections.

In addition to elucidating the HSPG extracellular interactome, a major challenge will be to determine whether, at the level of specific synaptic connections, differential HS modifications occur and are required for synapse development. By individual or simultaneous, cell type-specific, ablation of HS-modifying genes, it will be possible to explore whether HS modifications regulate different aspects of synapse-specific assembly and function. Altogether, these approaches will allow us to establish whether combinatorial codes of different HSPGs and specific HS modification exist, and whether such codes contribute to the specification of synaptic connectivity.

Finally, some members of the HSPG protein family, like GPC4, GPC5 and GPC6 have been linked to neurodevelopmental diseases such as autism, schizophrenia (Potkin et al., 2009; Doan et al., 2016). The link of glypicans to disease strengthens the importance of HSPGs in proper brain development and function. Therefore, elucidating the role of HSPGs and HS-modifying enzymes in the development of specific synaptic connectivity patterns will not only increase our understanding of the molecular logic underlying neural circuit assembly, but also provide new insight into the molecular basis of brain disorders.

## Author Contributions

GC and JW wrote the manuscript.

## Conflict of Interest Statement

The authors declare that the research was conducted in the absence of any commercial or financial relationships that could be construed as a potential conflict of interest.
